# The microbiological profile of lacrimal abscess: two decades of experience from a tertiary eye care center

**DOI:** 10.1186/1869-5760-3-57

**Published:** 2013-07-27

**Authors:** Mohammad Javed Ali, Swapna R Motukupally, Surbhi D Joshi, Milind N Naik

**Affiliations:** 1 Dacryology Service, Ophthalmic Plastics Surgery, L.V. Prasad Eye Institute, Banjara Hills, Hyderabad 500034, India; 2 Jhaveri Microbiology Service, L.V. Prasad Eye Institute, Banjara Hills, Hyderabad 500034, India

**Keywords:** Microbiology, Lacrimal, Abscess, Infection

## Abstract

**Background:**

The aim of this study is to exclusively report the microbiological spectrum of lacrimal abscess and the antibiotic sensitivity patterns of the organisms. Retrospective interventional study on 112 eyes of 112 patients who presented to the ophthalmic plastic clinic of a tertiary eye care center over a period of 23 years from January 1990 to February 2013 with lacrimal abscess were reviewed for demographic and microbiological profile. The culture results, organisms isolated, and their antibiotic sensitivity were studied.

**Results:**

The mean age at presentation was 37 years. The female to male ratio was 2:1. There was no significant difference in the laterality between the right and left eyes. Gram-positive organisms were the most commonly isolated accounting for 56.3% (63/112), and the commonest species isolated was *Staphylococcus aureus* in 25% (28/112) of the patients. *Hemophilus influenzae* was the commonest gram-negative isolate accounting for 30.2% of all the gram-negative isolates. Of the patients, 10.7% (12/112) showed no organisms on smear as well as sterile cultures. Gram-positive organisms were commonly sensitive to penicillins and vancomycin whereas gram-negative organisms were sensitive to quinolones and aminoglycosides.

**Conclusions:**

Gram-positive organisms are quite common as compared to gram-negative ones in cases of lacrimal abscess. The results of this study have significant bearing on the treatment of patients with lacrimal abscess.

## Background

Acute dacryocystitis is an acute inflammation of the lacrimal sac secondary to nasolacrimal duct obstruction and possibly bacterial overgrowth in the stagnant fluid in the lacrimal sac. There is a varied spectrum of its clinical presentations ranging from tenderness and erythema of the overlying tissues to a frank lacrimal abscess [1-4]. Untreated lacrimal abscess can progress to orbital cellulitis, superior ophthalmic vein thrombosis, and cavernous sinus thrombosis [5-7].

Most articles in the literature have discussed the microbiologic spectrum of chronic dacryocystitis [2,8-17], and very few dealt with acute dacryocystitis [2,18-20]. There is yet a lacuna in the literature with regard to reporting the microbiological spectrum exclusively for lacrimal abscess with antibiotic sensitivity profiles. We hereby present the largest series till date from a tertiary eye care center.

## Methods

**Figure 1:**
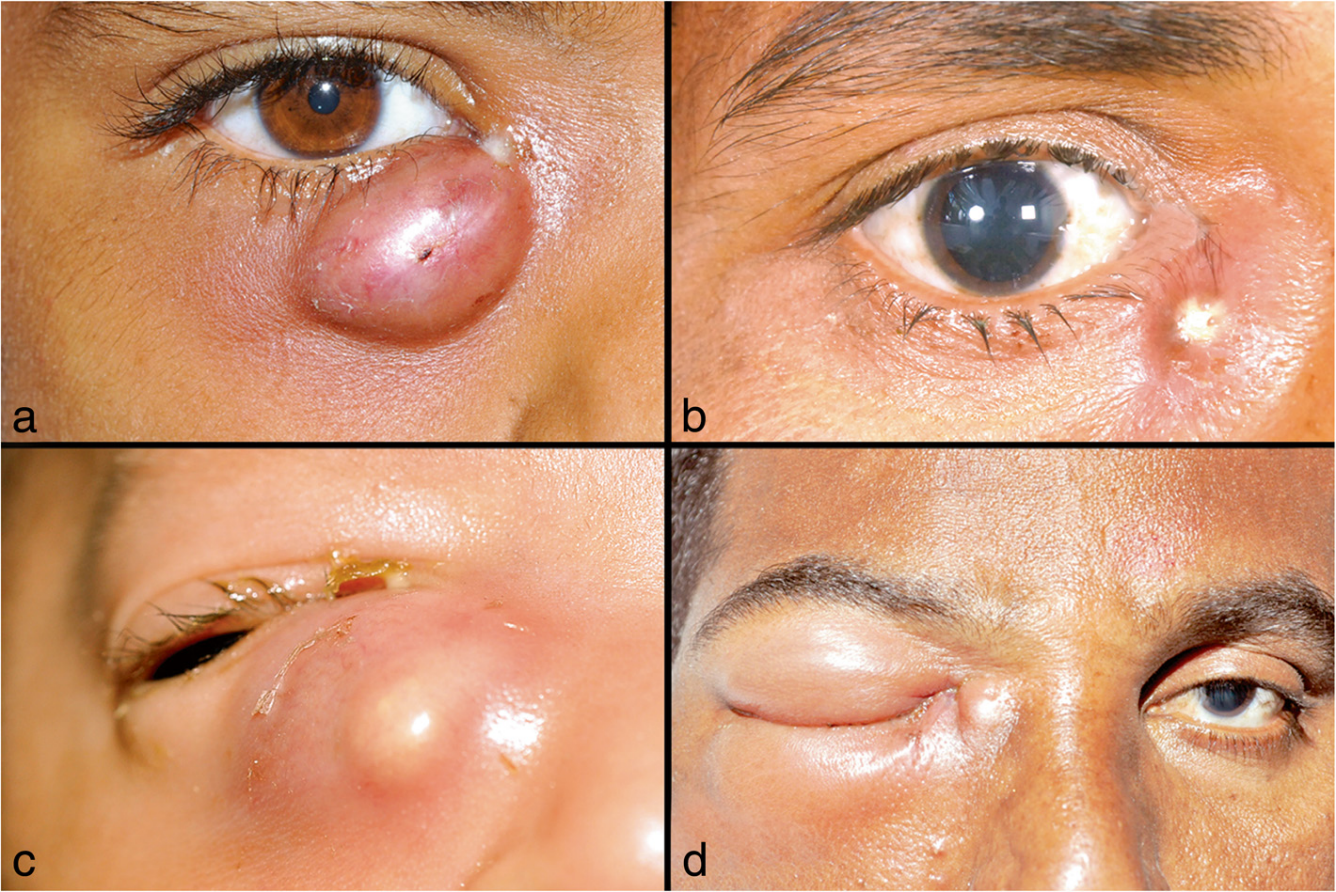
**Clinical spectrum of lacrimal abscess.** External photograph showing a well-localized right lacrimal abscess with discharge at the medial canthus **(a)**. Clinical photograph depicting the pus point of the abscess **(b)**. A neonate with right lacrimal abscess **(c)**. Lacrimal abscess with orbital cellulitis **(d)**.

**Figure 2:**
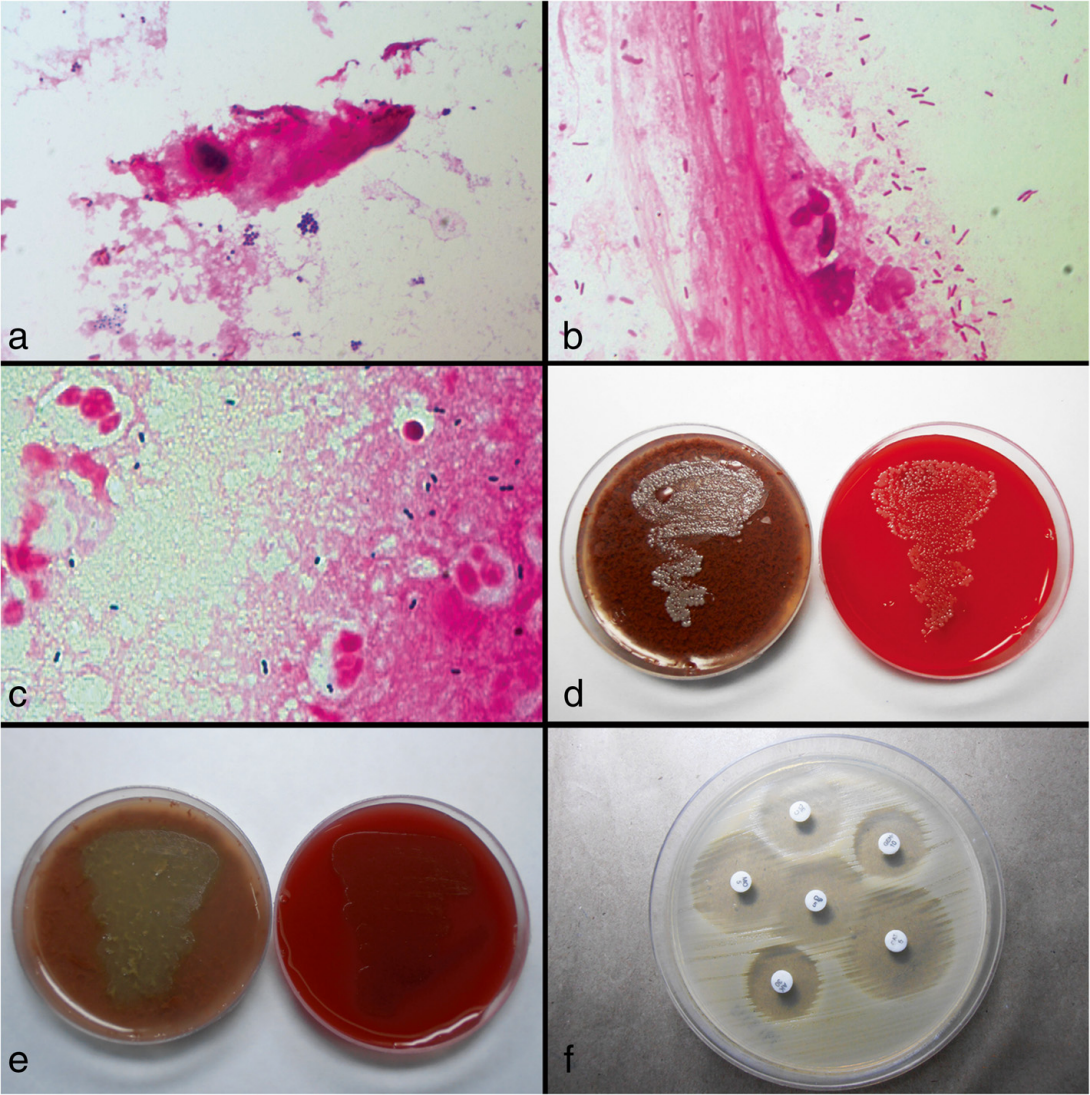
**Microbiological spectrum of lacrimal abscess.** Gram stain showing gram-positive cocci arranged in clusters, ×1,000 **(a)**. Gram stain showing plenty of gram-negative bacilli, ×1,000 **(b)**. Gram stain showing lanceolate gram-positive cocci arranged in pairs, suggestive of streptococcus, ×1,000 **(c)**. Culture plates of blood agar and chocolate agar showing confluent gray, moist, translucent growth **(d)**. Chocolate agar showing small, pinpoint colonies with a greenish discoloration and blood agar showing confluent alpha hemolytic growth **(e)**. Antibiotic susceptibility testing by Kirby-Bauer disk diffusion method **(f)**.

Medical records of patients diagnosed with lacrimal abscess, which underwent percutaneous drainage and the purulent material was sent for microbiological analysis from January 1990 through February 2013, were retrospectively reviewed. The study was carried out in accordance with the ethical guidelines of the Declaration of Helsinki, and institutional review board approval was taken. The diagnosis of lacrimal abscess was based clinically on the presence of a pus-filled lacrimal sac and perilacrimal tissues in a setting of an acute dacryocystitis (Figure 1a,b,c,d). Data retrieved include demographic profile, clinical examination findings, purulent material inoculation onto smears, and various culture media. None of the patients were using antibiotics at presentation. The culture results, organisms isolated, and their antibiotic sensitivity were studied (Figure 2).

Pus from lacrimal abscess was collected carefully avoiding any contact with the skin or the wound edges and inoculated directly onto two plates of 5% sheep blood agar, chocolate agar, brain-heart infusion broth (BHI), Sabouraud dextrose agar (SDA), and potato dextrose agar (PDA). One plate of blood agar was incubated under anaerobic conditions, while all other media were incubated aerobically. Blood agar, chocolate agar, and BHI are incubated at 37°C, while SDA and PDA are incubated at 25°C. The plates are observed for the presence of any growth daily up to 7 days. In case of positive cultures, the bacteria were further subjected to identification and antibiotic susceptibility testing. The bacterial isolates were identified using API system (Biomerieux, Marcy l'Etoile, France) or Vitek 2 Compact (Biomerieux, Marcy l'Etoile, France) automated identification systems.

Antibiotic susceptibility test was determined by the Kirby-Bauer disk diffusion method. Clinical and Laboratory Standards Institute guidelines were followed in performing the tests and interpretation. Antimicrobial disks commonly used included amikacin, gentamicin, tobramycin, ampicillin, amoxicillin, oxacillin, cloxacillin, cefazolin, ceftriaxone, cefepime, cefuroxime, ceftazidime, ciprofloxacin, ofloxacin, gatifloxacin, moxifloxacin, cotrimoxazole, chloramphenicol, vancomycin, piperacillin, ticarcillin, tazobactam, and imipenem.

The primary treatment offered to these patients with a lacrimal abscess was a percutaneous incision and drainage procedure followed by medical management in the form of broad-spectrum systemic antibiotics initially and anti-inflammatory agents like ibuprofen. The antibiotics were later modified as per sensitivity report, where needed. Inpatient care was provided to patients where injectable antibiotics were used as in the case of associated severe preseptal cellulitis or orbital cellulitis. Following resolution of the infection, the patients were scheduled for dacryocystorhinostomy.

## Results

A total of 112 eyes of 112 patients were enrolled for this retrospective study spanning over 23 years in duration from January 1990 through February 2013. The mean age at presentation was 37 years (range 22 days to 75 years). There were 70 (62.5%) females and 42 (37.5%) males. The female to male ratio was approximately 1.7:1. There was no preponderance of laterality. The right eye was affected in 53 (47.3%) patients and the left eye in 59 (52.7%) patients. Of the 112 patients, 13 (11.6%) were from the pediatric age group.

The most common organisms were gram-positive isolates, seen in 58.9% (66/112). Of the gram-positive isolates, 95.4% (63/66) were cocci and 4.6% (3/66) were bacilli. Overall, the gram-positive cocci contributed to 56.3% (63/112) of all the isolates, whereas 2.6% (3/112) were gram-positive bacilli. Gram-negative bacilli followed gram-positive cocci as the second most common isolates, seen in 30.4% (34/112) of all the isolates. There were no isolates of gram-negative cocci. Of the patients, 10.7% (12/112) did not show any organisms on smear, and the cultures were sterile as well. Table 1 summarizes the broad categories of isolates.

**Table 1:** Broad category of isolates identified

**Isolate type**	**Number (*n *= 112)**
Gram-positive cocci	63 (56.3%)
Gram-negative cocci	0 (0%)
Gram-positive bacilli	3 (2.6%)
Gram-negative bacilli	34 (30.4)
Sterile cultures	12 (10.7%)

Of the total 100 culture-positive cases, 15 different kinds of organisms and their species were isolated. The commonest organism isolated was *Staphylococcus aureus* in 28 patients (42% of all gram-positive isolates, *n* = 66), followed by *Streptococcus pneumoniae* in 17 patients (25.8% of all gram-positive isolates, *n* = 66) and *Hemophilus influenzae* in 11 patients (32.3% of all gram-negative isolates, *n* = 34). Other organisms isolated in a sizeable number of patients include *Staphylococcus epidermidis*, *Escherichia coli*, *Streptococcus pyogenes*, and *Pseudomonas aeruginosa*. Table 2 summarizes the details of the organisms isolated.

**Table 2:** Overall isolated organisms and their species

**Organism isolated**	**Number (*n *= 100)**
*Staphylococcus aureus*	28
*Streptococcus pneumoniae*	17
*Hemophilus influenzae*	11
*Staphylococcus epidermidis*	9
*Escherichia coli*	8
*Streptococcus pyogenes*	7
*Pseudomonas aeruginosa*	7
*Klebsiella pneumoniae*	4
*Corynebacterium diphtheriae*	2
*Streptococcus anginosus*	2
*Hemophilus parainfluenzae*	1
*Citrobacter diversus*	1
*Citrobacter freundii*	1
*Aeromonas salmonicida*	1
*Brevibacterium casei*	1

If we separately analyze the 13 pediatric patients in this group, there was no sex predilection with 7 males and 6 females. The mean age at presentation was 30.6 months (range 22 days to 108 months). The commonest organism isolated was *S. aureus* in 38.5% (5/13) patients. Table 3 summarizes the organisms isolated from the pediatric subset of this study group.

**Table 3:** Organisms isolated from the pediatric subset of the study group

**Isolate type**	**Number (*n *= 13)**
*Staphylococcus aureus*	5 (38.5%)
*Streptococcus pneumoniae*	3 (23%)
*Staphylococcus epidermidis*	1 (7.7%)
*Escherichia coli*	1 (7.7%)
*Staphylococcus epidermidis*	1 (7.7%)
*Pseudomonas aeruginosa*	1 (7.7%)
*Streptococcus anginosus*	1 (7.7%)

The common isolates, namely the *S. aureus* and *S. pneumoniae*, showed good sensitivity to penicillins, cephalosporins, and vancomycin. The other common gram-negative organisms showed good sensitivity to quinolone, cephalosporin, and aminoglycoside groups of antibiotics. Few rare isolates like *Streptococcus anginosus* and *Brevibacterium casei* were susceptible to all the antibiotics tested. Two isolates, one *S. aureus* and one *E. coli*, were multidrug resistant and susceptible only to higher penicillins like piperacillin and ticarcillin. No pandrug-resistant bacteria were isolated. Table 4 summarizes the commonly susceptible antibiotics found for different species.

**Table 4:** Commonly noted antibiotic sensitivity patterns

**Organism isolated**	**Commonly sensitive antibiotics**
*Staphylococcus aureus*	Pen/Ceph/Van
*Streptococcus pneumoniae*	Pen/Ceph/Van
*Hemophilus influenzae*	Quin/AMG/Chlo
*Staphylococcus epidermidis*	Pen/Ceph
*Escherichia coli*	Quin/Chlo
*Streptococcus pyogenes*	Pen/Ceph
*Pseudomonas aeruginosa*	Pen/Ceph/Quin
*Klebsiella pneumoniae*	Quin/AMG
*Corynebacterium diphtheriae*	Ceph/Van
*Streptococcus anginosus*	All tested
*Hemophilus parainfluenzae*	Quin
*Citrobacter diversus*	Quin
*Citrobacter freundii*	Pen/Quin
*Aeromonas salmonicida*	Pen/Ceph/Chlo
*Brevibacterium casei*	All tested

*Pen* penicillins, *Ceph* cephalosporins, *Van* vancomycin, *Quin* quinolones, *AMG* aminoglycosides, *Chlo* chloramphenicol.

## Discussion

Lacrimal abscess is a very painful condition whose pathogenesis is believed to be bacterial overgrowth in the stagnant fluid of the lacrimal sac in the background of nasolacrimal duct obstruction [1-4].

Most of the studies on the microbiological profile of dacryocystitis are on chronic dacryocystitis [8-17]. Very few of them deal with acute dacryocystitis [2,18-20], and only one discusses lacrimal abscess superficially [18]. The general trend in chronic dacryocystitis reflects culture-positive rates ranging from 52.5% to 97.3% with isolation rates of gram-positive organisms ranging from 53.7% to 75% and those of gram-negative organisms from 25% to 37.4% [8-14]. The most common gram-positive organisms isolated include *S. aureus* (worldwide), *S. pneumoniae* (Africa), and *S. epidermidis* (USA). Among the gram-negative isolates, there is a variable predominance like that of *H. influenzae* (Middle East), *P. aeruginosa* (North India and USA), *E. coli* (Europe), and *Corynebacterium diphtheriae* (China) [8-14]. Sun et al. [13] from China and Brook and Frazier [14] from USA, although reported *Staphylococcus* isolation as the most common, however, found fungus in 8% (*n* = 100) and 5% (*n* = 62) of their isolates, respectively. With *S. aureus* being the commonest isolate across most studies, focus has been on the increasing incidence of community-acquired methicillin-resistant *S. aureus* and the challenges it is likely to post in the future in terms of antibiotic resistance and treatment [15-17].

Microbiologic spectrum of acute dacryocystitis has been studied in 23 patients by the American Society of Ophthalmic Plastic and Reconstructive Surgery (ASOPRS) dacryocystitis group [19], and it was found that 78.3% of the isolates were gram-positive and 21.7% were gram-negative. Among the gram-positive isolates, *S. aureus* was the most common organism noted, accounting for 50% of all the gram-positive isolates. Among the gram-negative organisms, there was no preponderance of any organism with equal incidence of isolates of *P. aeruginosa*, *Fusobacterium*, and *Stenotrophomonas maltophilia*. The current study, though exclusively looks at lacrimal abscess, concurred with some of the findings of the acute cohort of the ASOPRS dacryocystitis group. Of our patients, 58.9% showed gram-positive isolates and 30.4% of the patients had gram-negative isolates. *S. aureus* accounted for 42.4% (28/66) of all the gram-positive isolates. However, unlike the ASOPRS group, our gram-negative profile was very different with *H. influenzae* and *E. coli*, accounting for 32.3% and 23.5% of all the gram-negative isolates, respectively (*n* = 34). Razavi et al. [20], in contrast to ASOPRS group findings, concluded that there are significant differences in the isolates between acute and chronic dacryocystitis, although the study did not show much difference, and the sample size of acute cases was only 12 patients. Huber-Spitzy et al. [2] studied both acute and chronic dacryocystitis and found 25% of their isolated overall to have gram-negative organisms, of which *E. coli* was the commonest (11.7%,17 cases). Briscoe et al. [18] exclusively studied acute dacryocystitis in 39 patients and found the predominance of gram-negative isolates (61%) with *P. aeruginosa* being the commonest isolate. However, only four of these patients had lacrimal abscess, and the details of isolates from these cases has not been mentioned separately.

The pediatric patients in the current study comprised only 11.6% of the entire study group, probably reflecting that the development of lacrimal abscess is not very common in congenital nasolacrimal duct obstructions. The microbiological profile was not found to be different in the pediatric subset of this study group with *S. aureus* being the most common organism followed by *S. pneumoniae.*

Limitations of our study includes slightly different disks used for antibiotic susceptibilities over a 23-year period, and this is not unusual and can happen based on evolving knowledge and experience. Referral bias could have contributed to isolation of rare organisms.

## Conclusions

In conclusion, we presented the largest series and exclusive microbiological profile of lacrimal abscess. The evolving profile of microorganism and their sensitivity patterns give us an insight into newer challenges of antibiotic resistance and appropriate treatment options.

## Competing interests

The authors declare that they have no competing interests.

## Authors’ contributions

AMJ conceived of the study and wrote the manuscript. MSR participated in the microbiology work. JSD performed the data analysis. NMN participated in the critical review. All authors read and approved the final manuscript.
